# Transcriptome Reprogramming in Heart Failure: The Hidden Splicing Code

**DOI:** 10.1007/s11886-026-02386-0

**Published:** 2026-06-27

**Authors:** Francisca Akhigbe, Ningjing Song, Jeyashree Alagarsamy, Haobo Li, Chen Gao

**Affiliations:** 1 Department of Pharmacology, Physiology and Neurobiology, University of Cincinnati, Cincinnati, OH USA; 2 Department of Pathology, University of Cincinnati, Cincinnati, OH USA; 3 Department of Anesthesiology, Massachusetts General Hospital, Boston, MA USA; 4 Harvard Medical School, Boston, MA USA

**Keywords:** Alternative splicing, Heart failure, Cardiomyopathy, Cardiometabolic disorder, Therapeutics

## Abstract

**Purpose of Review:**

Heart failure remains a major cause of morbidity and mortality that is associated with myocardial changes in metabolism, contractile function, and molecular remodeling. Cardiomyopathies comprise a diverse group of disorders that can be triggered by various external and internal stressors. This review aims to cover the underlying molecular mechanism driving heart failure progression, at the level of alternative splicing.

**Recent Findings:**

Alternative splicing is a fundamental mechanism that expands transcriptomic diversity through the differential inclusion or exclusion of exons. This process enables a single gene to generate multiple mRNA isoforms, thereby fine-tuning gene function in a context-dependent manner. Splicing outcomes are determined by a highly coordinated regulatory network, including cis-acting splicing elements, transcriptional kinetics, and trans-regulatory RNA-binding proteins, which together form a dynamic “splicing code” that responds to physiological and pathological stresses. In the heart, alternative splicing regulates cardiac cell homeostasis and normal physiological function. Dysregulated alternative splicing has been increasingly recognized as a key contributor to cardiovascular diseases, particularly in the context of sarcomere gene isoform switching. However, emerging evidence suggests that cardiomyopathies arising from distinct etiologies including dilated, ischemic, and cardiometabolic disorder are associated with unique splicing programs.

**Summary:**

Here, we provide a comprehensive overview of the regulatory mechanisms governing alternative splicing in the heart, with a particular emphasis on disease-specific splicing events across different forms of cardiomyopathy. We further discuss recent advances in targeting aberrant splicing for therapies as well as novel splicing analysis platforms, highlighting the potential of RNA-based strategies to modulate splicing in heart failure.

## Introduction

In response to diverse stresses such as genetic mutations, hypertension, and coronary disease, the heart initially adapts to preserve cardiac function; however, failure of these compensatory mechanism drives a transition to heart failure, marked by cardiomyocyte remodeling, cell death, and fibrosis [1–3]. Cardiomyopathies comprise a diverse group of disorders with distinct impacts on cardiac function as well as metabolic and molecular remodeling, with major subtypes including dilated cardiomyopathy (DCM), ischemic cardiomyopathy (ICM), and cardiometabolic disease-associated cardiomyopathy [4–7].

Alternative splicing in eukaryotic genes significantly increased transcriptome diversity through differential exon utilization. This process enables a single gene to generate multiple mRNA isoforms with distinct and sometimes opposing functions [8]. In the heart, alternative splicing is a key post-transcriptional regulatory process with a critical role in cardiac function and physiology [9].

## Alternative Splicing: A Novel Stress Sensor in Cellular Physiological and Pathological Responses

### Alternative Splicing Definition, Prevalence, and Contribution to Eukaryotic Transcriptome Complexity

The discovery of “split genes”, which was later defined as RNA splicing, fundamentally reshaped our understanding of gene expression [10]. Eukaryotic genes are initially transcribed into mRNA precursors (pre-mRNA) containing introns that must be spliced out so that the exons can be ligated together. This process, termed mRNA splicing, is catalyzed by spliceosome, a highly coordinated ribonucleoprotein complex [11]. Splicing reaction is initiated by the U1 and U2 small ribonucleoproteins (snRNPs), which mark an intron and recruit the U4/U6.U5 tri-snRNP. The 5’ splice site (5’SS) is transferred from U1 to U6 snRNA, leading to the re-arrangement of U6 and U2 snRNA, generating an RNA-based active site, with 5’SS positioned at two catalytic metal ions prior to be attacked by the branch point (BP) adenosine. 3’ splice site (3’SS) binds and removes the BP adenosine, after where the 5’ free exon attacks the 3’SS to generate mature mRNA along with an intron lariat [12]. Alternative splicing allows the different combinations of exons from a single mRNA precursor to be joined together, enabling one gene to generate multiple distinct mature mRNA and ultimately different protein isoforms with distinct or even opposing functions [13, 14].

In addition to spliceosome, the alternative splicing is regulated by the combination of cis-regulatory elements located within the pre-mRNA and trans-acting RNA-binding proteins to determine the exon inclusion or exclusion [15]. The concept of RNA splicing which was originally defined in 1977, has been shown to be one of the most important mechanisms regulating eukaryotic transcriptome complexity [10, 16, 17]. From early exon junction microarrays described by Johnson et al., which estimated that ~ 70% of human multi-exon genes are alternatively spliced, [18] to the development of RNA-sequencing technologies revealing that this proportion exceeds 90%, [14, 19] alternative mRNA splicing stands as a key mechanism explaining the contrast between the limited number of protein-coding genes in the human genome and the high functional complexity of higher organisms.

Alternative splicing exhibits strong tissue specificity, with distinct splicing programs associated with different organs and cell types. Alternative splicing is also highly dynamic in response to different physiological and pathological stresses. For example, in the landmark discovery by Wang et al., 15 diverse human tissues and cell lines were analyzed at the level of gene and mRNA isoform expression. It was found that mRNA isoforms expression differs significantly between tissues and that extreme, or “Switch-like” regulation of tissue-specific splicing is correlated with increased sequence conservation in regulatory regions [14]. It is worth noting that tissue-specific alternative splicing is conserved across species, but with species-specific adaptations. This has been shown in a comparative study in which alternative splicing profiles across four mammals and one bird were compared among nine tissues. It was concluded that while gene expression programs are largely conserved at the tissue level, alternative splicing, in contrast, is conserved only in a subset of tissues and is often lineage-specific [20]. In addition to tissue and cell type specificity, alternative splicing also contributes towards transcriptome remodeling in diseases and splicing defects are linked to numerous human diseases. In a comprehensive analysis of alternative splicing events across human cancer patients, it was discovered that tumors exhibit up to 30% more alternative splicing events than normal samples [21]. In another study in which neural-regulated alternative splicing events were profiled, the authors concluded that 3-27nt microexon are highly evolutionarily conserved and neuronal specific, and that these microexons are frequently mis-regulated in the brains of individual with autism [22].

### mRNA Alternative Splicing as a Stress Sensor

Alternative splicing has emerged as a sensitive regulatory layer that responds to cellular stress, including hypoxia, metabolic changes, and inflammation.

Hypoxia is a fundamental stress in both physiological and pathological contexts, including development, ischemic injury, and tumor progression. While the transcriptional regulation orchestrated by hypoxia-inducible factors (HIFs) during the hypoxia response has been well documented, [23, 24] emerging evidence suggest that post-transcriptional regulation, particularly alternative splicing, plays a critical role in fine-tuning the hypoxic response. In tumor, hypoxia induces widespread changes in alternative splicing across different cell types [25]. It is suggested that hypoxic stress changes genome-wide exon inclusion, intron retention and splice site selection [26]. Functionally, hypoxia driven splicing has been shown to impact alternative splicing of vascular endothelial growth factor (VEGF), generating distinct isoforms with different biological activities, thereby altering the functional outcome of angiogenesis [27].

Alternative splicing is also a regulatory mechanism that rewires metabolism under stress. A classic example is the PKM1-PKM2 splicing isoform switch, a key regulator of aerobic glycolysis in cancer cells [28]. Beyond PKM, studies have further revealed that alternative splicing actively shapes metabolic pathways, including mitochondrial function, lipid and glucose metabolism [29]. In addition, metabolic stress, including nutrient deprivation and energy imbalance, are also shown to impact on splicing outcomes through metabolic signaling pathways including mTOR, thereby linking cellular energy status to post-transcriptional regulation [30]. For example, activation of mTORC1 leads to downstream signaling S6 kinase (S6K) activation and further phosphorylates splicing factors, leading to changes in splice site selection and exon inclusion levels. This underscores the importance of post-transcriptional regulation in metabolic adaptation [31].

Inflammation represents another major stress condition under which alternative splicing plays a critical role. In immune cells, alternative splicing contributes to cell-type-specific response by regulating cytokine production and receptor signaling. For example, a genome-wide study investigating alternative splicing events in macrophages from lipopolysaccharide treated mice has identified substantial alternative mRNA splicing events. These inflammation-induced changes in alternative splicing events are mainly enriched in chemokine signaling and cellular metabolism [32]. Functionally, alternative splicing can mediate immune signaling through generating different isoforms in inflammatory response. One example is the alternative splicing of MyD88, the shorter isoform of MyD88 serves as negative regulator of NF-kB activation, providing a feedback mechanism to overcome excessive inflammation [33, 34]. A second example is the Ca^2+^/calmodulin-dependent kinase II (CaMKII) splicing in the heart, where the splicing variants, CaMKII-δ9 and CaMKII-δ3 inversely regulate cardiomyocytes viability and inflammation response through interacting with NFkB inhibitor a (IkBα) [35].

Together, these findings highlight that alternative splicing acts as an active regulator for cellular stress responses, ranging from hypoxia and metabolic stress to inflammation.

### Alternative Splicing Regulation: Timing, Code, and Regulators

Given the dynamic landscape of alternative splicing events in different tissue types and in response to disease status, it is not surprising that althernative splicing is a highly coordinated process that involves transcription kinetics, cis-regulatory elements within the pre-mRNA, and trans-regulatory splicing regulators.

Co-transcriptional splicing is a dominant form of splicing in eukaryotes that introns are removed while RNA is still being transcribed by RNA polymerase II (Pol II). This is achieved through Pol II transcription elongation complex making contacts with the splicing machinery, allowing splicing to occur once the 3’ splice site has been synthesized [11]. During co-transcriptional splicing, RNA Pol II elongation speed influences splice site recognition and exon splicing. Slower transcription can promote alternative exon inclusion by allowing more time for spliceosome assembly, where rapid elongation usually leads to exon skipping [36, 37].

Within the pre-mRNA, the concept of “splicing code” defines the RNA features and regulatory sequences that determine the tissue-specific splicing profiles [15]. Within mRNA, the splicing code includes splice sites, branch points, and polypyrimidine tracts that facilitate recognition by the spliceosome, as well as splicing enhancers and silencers that recruit RNA-binding proteins to regulate exon inclusion or exclusion [38]. This “Splicing code” concept is further refined by using computational analysis approaches in large transcriptomic datasets. In fact, the computational based predictive models have been developed to predict the splicing outcomes through incorporating large amount of sequence features, including splice site strength, exon and intron length as well as regulatory motif distribution. This has been demonstrated in human splicing patterns where the genomic sequence features can be used to predict the splicing decision, pointing to a highly coordinated regulatory code for alternative splicing [39]. Importantly, these models allow the prediction of how genetic variants impact on splicing outcome, paving the path to link genomic variation to disease-associated splicing events [39].

While the splicing code provides a regulatory blueprint, the final splicing outcome is executed by the trans-acting RNA-binding proteins, or splicing factors. Classic splicing regulators include serine/arginine-rich (SR) proteins, which are generally considered to promote exon inclusion, and heterogeneous nuclear ribonucleoproteins (hnRNPs), which often repress exon inclusion (splicing repressors). In addition, numerous other RNA-binding proteins, including RNA binding protein, fox-1 homolog (RBFOX), Muscleblind-like (MBNL), and PTBP proteins, coordinate to regulate alternative splicing in a context dependent manner. Recent advances in proteomics analyses have further raised the possibility that human proteome contains a diverse repertoire of RNA binding proteins that remain largely incompletely characterized [40]. For example, Castello et al. reported the discovery of hundreds of RNA binding proteins within the human proteome, and a substantial proportion of these proteins lack classical RNA-binding domains, suggesting that the RNA-binding protein universe is far more extensive than previously appreciated [41]. The expression levels and activities of these splicing factors are highly tissue- and cell type-specific. Distinct expression profiles of RNA binding proteins across tissues and cell types contribute to unique splicing programs that ultimately define cellular identity. This further enables precise control of alternative splicing dynamics in response to developmental and physiological conditions. Indeed, changes in the expression of splicing factors have been implicated in a broad range of human diseases, including cancer, neurological disorders, and muscular diseases, highlighting the critical role of splicing factor regulation in cellular physiology [42–47].

Among these, several RNA binding proteins have been implicated in playing important roles in heart. The RBFOX protein family members regulate alternative splicing through recognition of the conserved (U)GCAUG motif and have enriched expression pattern in both neuronal and muscle tissues. Depending on the position of the binding sites relative to target exons, RBFOX proteins can either promote or inhibit the exon inclusion, highlighting position-dependent regulation of splicing [48, 49]. MBNL family is another essential splicing regulator family that has been shown to regulate developmental splicing dynamics in muscle. MBNL family members are also known for their roles in myotonic dystrophy where splicing defects are caused by the sequestration of MBNL proteins [50]. Lastly, RBM20 controls the splicing of sarcomere genes, most notably the giant protein titin (TTN), thereby directly influences cardiac contractile function [51].

## Alternative Splicing in Heart Failure

Among different tissues, the heart is a unique system in which fine-tuned regulation of alternative splicing is essential for its function and physiology. The contractile function and metabolic dynamics of cardiomyocytes require highly regulated gene expression at both the transcriptional and post-transcriptional levels, and even subtle changes in splicing can have profound impact on cardiac function. Indeed, mis-regulated alternative splicing events have been directly linked to different forms of cardiomyopathy.

### Alternative Splicing Events in Dilated Cardiomyopathy

Dilated cardiomyopathy (DCM) is characterized by ventricular dilation and systolic dysfunction, resulting in impaired cardiac output and ultimately heart failure with high mortality. While transcriptional remodeling has been recognized as a key molecular event during DCM reprogramming, emerging studies have shown that post-transcriptional regulation at the level of alternative splicing, plays an important role in disease progression. Genome-wide analysis in both human patients and rodent models have shown large-scale alterations in alternative splicing profiles in DCM [52, 53]. DCM exhibits a clear connection between alternative splicing and sarcomere dysfunction, particularly through the RNA binding motif protein 20 (RBM20)-dependent splicing of TTN. RBM20 plays a crucial role in regulating the balance of TTN isoforms; when its activity is disrupted, it can lead to a shift toward longer, more compliant TTN isoforms. This alteration reduces passive stiffness, which in turn contributes to ventricular dilation and systolic dysfunction [54]. Additionally, splicing of Tropomyosin 1 (TPM1) may also play a role in this process. Increased expression of the alternatively spliced TPM1-K isoform has been associated with reduced myofilament tension and the induction of a DCM-like phenotype [55].

Recent advances in sequencing technologies have further revealed dynamic changes in splicing factors and splicing events beyond sarcomere genes. For example, the alternative splicing of CaMKIIδ generates different splicing isoforms with different functions, mis-regulated CaMKIIδ splicing has been associated with impaired calcium handling in cardiomyopathy [56]. Mechanistically, cardiac enriched splicing factors, including ASF/SF2, as well as RBM20 have been implicated in regulating CaMKIIδ alternative splicing in heart [57, 58].

Changes in splicing factors expression or mutations within splicing factors have also been shown to impact cardiac function in DCM. Members of the RBFOX family, particularly RBFox1 has been implicated in cardiac splicing regulation, including cardiac transcription factor- MEF2 family members [59]. Similarly, RNA-Binding Protein With Multiple Splicing (RBPMS) is essential for maintaining adult cardiac function. Its loss results in widespread splicing alterations in contractile genes such as *Pdlim5* and *Nexn*, leading to ventricular dilation and systolic dysfunction, consistent with DCM [60].

### Alternative Splicing Events in Ischemic Cardiomyopathy

Ischemic cardiomyopathy (ICM) represents a clinically distinct form of heart failure defined by significant ventricular dysfunction and structural remodeling resulting from a persistent imbalance between myocardial oxygen supply and demand [61]. ICM is defined by the loss of viable cardiomyocytes and their replacement with non-contractile fibrotic scar tissue, which triggers a cascade of maladaptive mechanical and molecular responses [62]. While DCM has provided important insights into the role of alternative splicing in the heart, ICM exhibits both overlapping and distinct splicing alterations. Similar to DCM, genome-wide analyses have revealed a noticeable reduction in mRNA splicing efficiency in ICM, mainly affecting key sarcomere genes, including TTN [63]. Early microarray studies identified a significant decline in splicing efficiency in the hearts of ICM. Specifically, four core sarcomere transcripts- cardiac troponin T (TNNT2), cardiac troponin I (TNNI3), β-myosin heavy chain (MYH7), and Filamin C (FLNC)-exhibited substantial alterations, a pattern similar to that observed in DCM and aortic stenosis. These changes are characterized by an increase in intron retention events that result in the introduction of premature termination codons, which likely activate the nonsense-mediated decay pathway and ultimately dimmish functional protein production [63, 64].

ICM is characterized by cardiomyocyte death, inflammation, and subsequent scar formation, leading to profound cellular heterogeneity within the myocardium. Recent multi-chamber analyses indicate that ICM displays distinct spatial heterogeneity, with splicing alterations predominantly localized to peri-infarct regions of the left ventricle, highlighting regional vulnerability in response to ischemic injury [65]. Splicing regulation in cardiac fibroblasts serves as a major driver of post-ischemic remodeling. For example, fibroblast-specific ablation of RNA binding protein RBMS1 improves cardiac dysfunction by mitigating myocardial fibrosis through regulating the alternative splicing of LIM domain 7 (LMO7) [66]. In parallel, isoform switching of pyruvate kinase from *Pkm1* to *Pkm2* has been shown to regulate the transition from adaptive repair to pathological remodeling and fibrosis following myocardial infarction [67].

### Alternative Splicing Events in Cardiometabolic Disorder

The increasing prevalence of metabolic diseases, including diabetes and obesity, has been associated with a concurrent rise in cardiometabolic conditions such as diabetic cardiomyopathy (DiaCM) and heart failure with preserved ejection fraction (HFpEF) [68].

Heart failure resulting from DiaCM is a leading cause of mortality among individuals with diabetes, presenting with diastolic and systolic dysfunction, cardiac hypertrophy, and fibrosis [69]. Although the molecular mechanisms underlying DiaCM pathogenesis are not fully understood, recent studies have explored the role of alternative splicing in disease development. Chronic activation of protein kinase C (PKC) isoforms α and β has been implicated in DiaCM pathogenesis. In Type 1 diabetic mouse hearts, PKCα/β activation upregulates CELF1 and RBFOX2, leading to widespread splicing alterations [70]. Overexpression of CELF1 in adult mouse hearts is associated with splicing defects and progression to heart failure [70–72]. CELF1 CLIP-seq analysis identified aberrant exon exclusion in approximately 72% of CELF1 targets in the diabetic hearts, with cassette exon splicing events being the most common [71]. Reduced CELF1 and RBFOX2 levels and splicing activities are linked to these diabetes-induced splicing changes [71, 73.

Abnormal calcium handling in early DiaCM contributes to the development of cardiac diastolic dysfunction [74, 75]. RBFOX2-mediated alternative splicing regulation has been linked to genes involved in cGMP-PKG signaling pathways, including the L-type Calcium Channel (LTCC) [75, 76]. In diabetic hearts, mis-splicing of genes such as MEF2a, Atp2b1, and Cacna1 disrupts the cGMP-PKG-Ca^2+^ pathway, leading to impaired calcium homeostasis [75, 76]. In addition, PTBP1, an RNA binding protein that typically represses splicing during neuronal and cardiac developmnt [77], is implicated in DiaCM [78]. A diabetes-induced PTBP1 splice isoform (lacking exon 8 in mice and exon 9 in humans) in the adult heart exhibits reduced splicing repressive function [78, 79]. Reduced expression of the splicing factor SRSF3 in adult myocardium following myocardial infarction leads to excessive alterations in alternative splicing, resulting in severe cardiac dysfunction [69, 80]. Notably, SRSF3 overexpression in the DiaCM mouse heart prevents cardiomyocyte apoptosis and improves cardiac function [69].

HFpEF is a complex, multi-organ disorder characterized by diastolic dysfunction and impaired left ventricular filling, primarily due to increased left ventricular passive stiffness. Approximately 70% of this left ventricular passive stiffness is attributed to TTN-regulated cardiomyocytes [81, 82]. TTN, a large myofilament protein, generates passive force as sarcomeres stretch and facilitates the return of the sarcomere to its resting length. Changes in TTN alternative splicing has been identified as a significant contributor to diastolic dysfunction in HFpEF [83–85]. In the adult heart, alternative splicing of TTN is regulated by splicing factor RBM20, generating two major titin isoforms, a larger N2BA (~ 3.3 MDa) isoform and a shorter N2B (~ 3.0 MDa) isoform, which confers greater stiffness [51, 84, 86–88]. Complete inhibition of RBM20 in mouse hearts results in expression of a very large, highly compliant TTN isoform, N2BA-G (~ 3.9 MDa), which alters cardiac filling [89]. Partial inhibition of RBM20 induces expression of the N2BA-N TTN isoform (~ 3.5 or 3.6 MDa), leading to decreased left ventricular stiffness, preserved baseline systolic function, and improved exercise tolerance [87, 89].

**Fig. 1:**
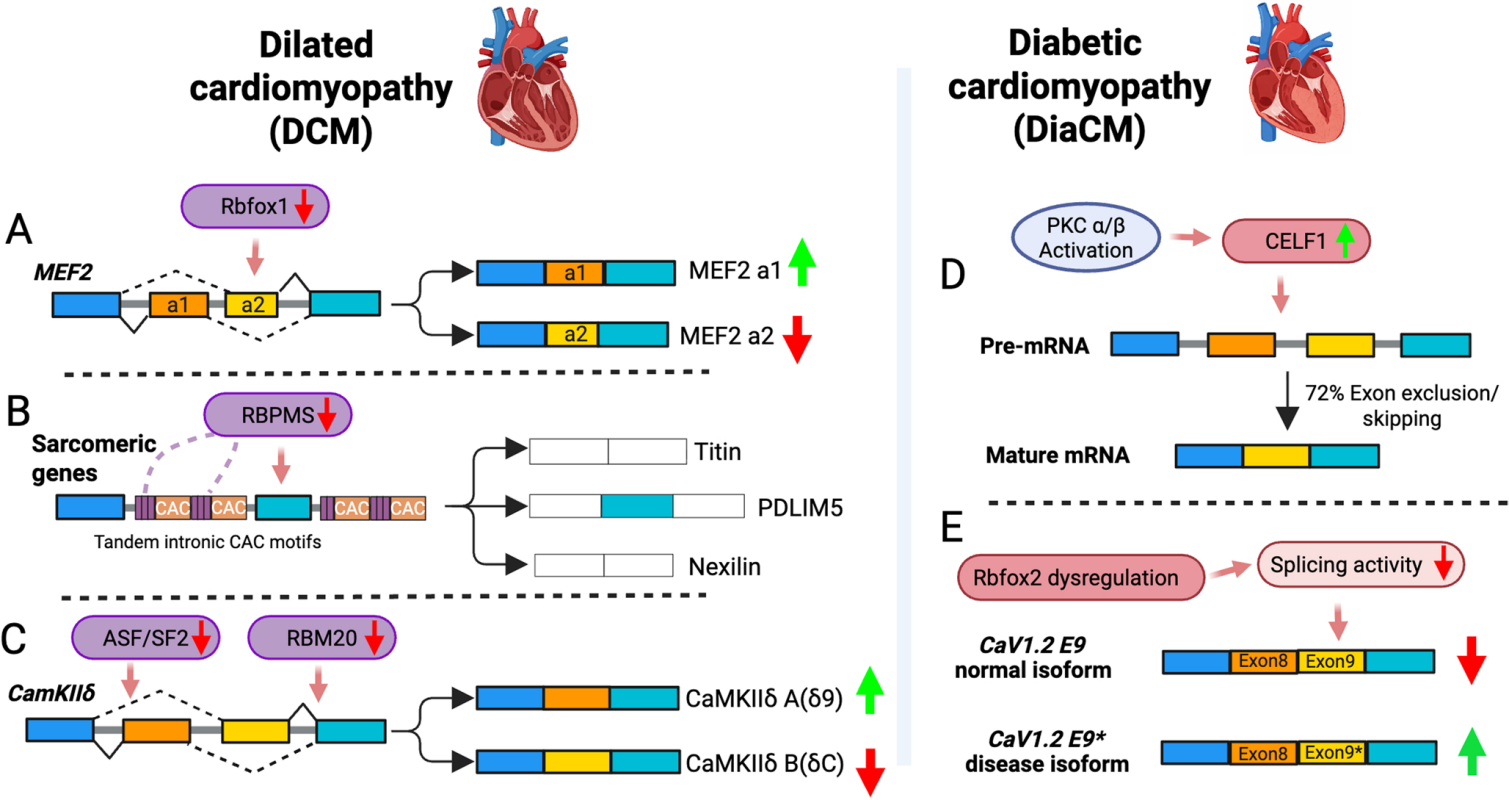
Schematic representation of the different types of alternative splicing and their effect on the mature mRNA product in dilated cardiomyopathy (DCM) and diabetic cardiomyopathy (DiaCM). Different colored boxes represent different exons. *Dot lines* indicate alternative splicing. RNA Binding Fox-1 Homolog 1 (Rbfox1), RNA Binding Protein, MRNA Processing Factor (RBPMS), Splice factors RNA-binding protein 20 (RBM20), and Serine/arginine-rich splicing factor 1 (SRSF1, renamed ASF/SF2) are shown in *long rounded rectangle of purple*. CUGBP Elav-Like Family Member 1 (CELF1) and RNA Binding Fox-1 Homolog 2 (Rbfox2) are shown in *long rounded rectangle of light red*. Protein Kinase c (PKC) is shown in *oval of light blue*. (**A**). Downregulation of RBFOX1 alters splicing process of transcription factor MEF2 family members that yields different MEF2 isoforms with differential effects on cardiac hypertrophic gene expression in pathological cardiac hypertrophy. This illustration is based on findings from Gao et al. [59] (**B**) RBPMS regulates mRNA alternative splicing of genes associated with sarcomere structure and function through recognizing tandem intronic CAC motifs, such as Ttn, Pdlim5, and Nexn, generating new protein isoforms in DCM. This illustration is based on results published in Gan et al. [60] (**C**) Cardiomyocytes deficient in ASF/SF2 and RBM20 results in a splicing switch of the Ca(2+)/calmodulin-dependent kinase IIδ (CaMKIIdelta) transcript with increased CaMKIIδA (δ9) and decreased CaMKIIδB (δC). This illustration is based on results published in Xu et al. and Maeda et al. [57, 58] (**D**) PKCα/β activation induces CELF1-mediated aberrant exon exclusion/skipping during pre-mRNA processing. 72% of CELF1 targets displayed aberrant exon exclusion in type 1 diabetic (T1D) hearts. This illustration is based on findings from Belanger et al. [71] (**E**) Dysregulation of RBFOX2 inhibits alternative splicing activity and upregulates CaV1.2 E9* isoform over the canonical CaV1.2 E9 isoform in DiaCM. This illustration is based on results published in Nutter et al. and Li et al. [73] Created in BioRender. Song, N. (2026) https://BioRender.com/u2wddqm

Given the differential impact of distinct etiologies on alternative splicing programs, targeting splicing isoform switching or key splicing regulators represents an attractive therapeutic strategy for precision treatment of cardiomyopathy (Fig. 1).

## Therapeutic Targeting Alternative Splicing in Heart Failure

### Targeting RNA-Binding Proteins as Heart Failure Therapy

Given the important role of RNA-binding proteins (RBPs) in regulating alternative splicing, targeting these RBPs represents an effective strategy to modulate alternative splicing programs in cardiac physiology and disease, particularly RBPs with known function in cardiac pathological remodeling, including the RBFOX, RBM20 and MBNL families.

In the heart, both RBFox1 and RBFox2 are down-regulated in heart failure, and RBFox1 regulates the alternative splicing of genes essential for cardiac hypertrophy and myocardial infarction [59, 90, 91]. Transgenic expression of RBFox1 in the heart improves cardiac function following pressure overload-induced DCM, and adeno-associated virus (AAVs) mediated RBFox1 gene delivery in rats has been shown to protect the heart from myocardial infarction-induced heart failure [59, 90].

Similarly, as a key regulator for TTN splicing, RBM20 mutations have been associated with DCM [51, 92]. Modulating RBM20 activity via genetic manipulation and antisense oligonucleotides (ASOs)-based therapy has been shown to improve cardiac compliance by altering TTN splicing isoform expression [93, 94]. Compared with targeting individual splicing events, modulation of RBPs offers coordinated reprogramming of splicing profiles that drive cardiac disease progression. However, targeting RBPs as a therapeutic strategy remains challenging due to their broad target specificity. Future research will require cell-type-specific delivery or modulation of RBPs activity to improve the therapeutic outcomes.

### Targeting Splicing Variants as Heart Failure Therapy

Therapeutic strategies are being actively developed to directly target specific splicing variants using sequence-based approaches, including AAVs-mediated delivery of splicing variants and ASOs-based splice site modulation.

Viral vector-based gene delivery is a well-established strategy to manipulate gene expression [95–97]. In the heart, AAV systems, an FDA approved gene therapy approach, can be used to achieve efficient and long-term expression of specific splicing variants and may serve as a therapeutic strategy to restore or induce desired splicing isoform expression in the dysfunctional heart [98]. For example, expression of the sarcomere protein isoforms, including dystrophin and myosin-binding protein C (cMyBP-C) has been shown to influence myocardial stiffness and cardiac performance [99, 100]. However, caution should be taken that AAV-mediated gene delivery can alter total gene expression rather than selectively switching endogenous alternative splicing outcome. As a result, AAV-mediated therapy might lead to non-physiological expression of the target genes instead of altering the splicing isoform ratio of the desired target.

**Fig. 2:**
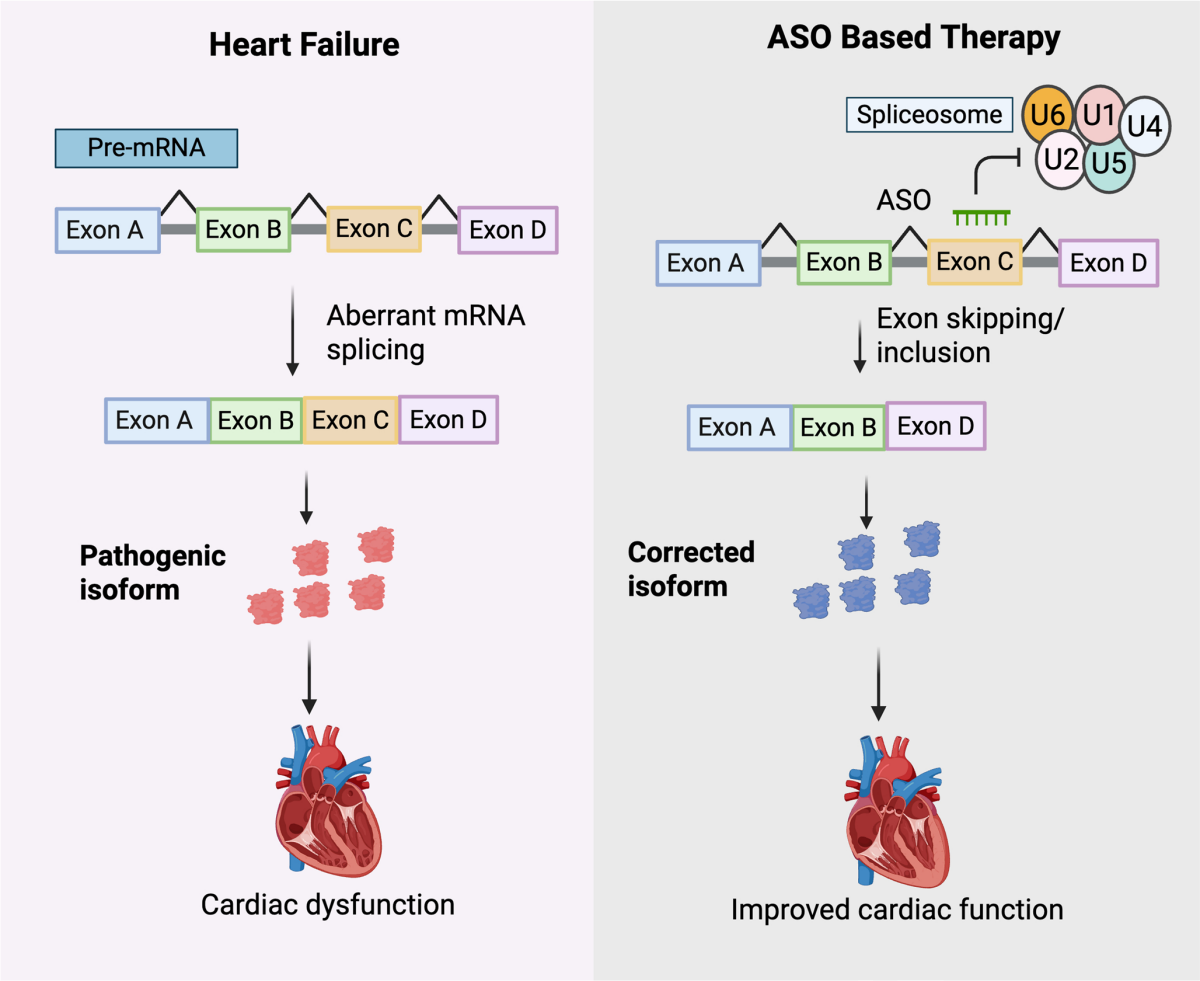
Summary of antisense oligonucleotides (ASO)–based therapy in heart failure. Created in BioRender. Alagarsamy, J. (2026) https://BioRender.com/stijojp

ASOs-based splicing modulation, aimed at either promoting or inhibiting exon inclusion, has been validated in multiple genetic disorders and is exemplified by FDA-approved therapies [101, 102]. In the cardiovascular field, ASO-based therapies are also being used to modulate splicing of genes involved in cardiac function. An important proof-of-concept approach in cardiovascular disease is modulating TTN splicing. This strategy aims to alter TTN isoform composition, which has been shown to play important roles in myocardial function. ASO-based approaches have been used to promote exon inclusion in TTN, resulting in increased expression of the longer isoform (N2BA, more compliant isoform) and thereby improving diastolic function in preclinical models [103, 104]. Furthermore, in our recent studies, we demonstrated that muscleblind like protein 1 (Mbnl1) undergoes a splicing switch following myocardial infarction, characterized by selectively including a 36nt microexon. Targeted blockade of this microexon inclusion using ASOs can protect the mouse heart from myocardial infarction-induced heart failure by reducing cardiomyocytes cell death [90]. By selectively switching isoform expression, ASO-based therapeutic strategy offers the option to fine-tune protein function without changing total gene expression levels (Fig. 2).

Together, these strategies highlight the therapeutic potential of targeting alternative splicing at multiple levels, from global regulatory factors to individual transcript isoform.

## Future Directions: Technology Advances and Biological Scope

Accumulating evidence supports a new model in which alternative splicing is a dynamic regulatory layer that integrates diverse stress signals, including hypoxia, metabolic perturbations, and inflammation, to fine-tune gene expression programs. The coordinated regulation of splicing factors serves as a central component of cardiac physiology and pathological remodeling.

### Technology Advances

Looking forward, technological advances will drive the next phase of splicing research. Recent studies have demonstrated the capacity of single-cell RNA sequencing, including SCASL (single-cell clustering based on alternative splicing landscapes), in detecting splicing events at cellular resolution and revealing splicing heterogeneity across different cell types and states [105, 106]. In parallel, long-read RNA sequencing and Nanopore technologies enable direct reading of full-length transcripts, overcoming limitations of traditional short-read sequencing and read assembly, with sustantially improved resolution of splicing complexity [107–109]. In addition, the integration of these approaches with cutting-edge spatial transcriptomics and proteomics platforms offers detailed splicing maps within the native tissue context [110, 111]. Such multi-omics approaches will enable the identification of cell-type-specific splicing programs under spatial resolution, as well as prediction of their functional consequences at the protein level.

Beyond the splicing and sequencing platforms, emerging integrative computational analyses have further enabled the prediction of the splicing code, linking sequence features and RBPs to reveal context-dependent splicing outcomes. Several novel computational approaches have been developed to overcome the challenge of accurately quantifying exon inclusion levels under limited sequencing depth. For example, Single-Cell Splicing Estimation (SCSES) uses data diffusion to infer missing splicing information by sharing data across similar cells and events, thereby improving Percent Spliced-In (PSI) calculations [105]. Similarly, Bayesian-based methods, including BRIE, leverage prior information and read distribution patterns to infer PSI values at the single-cell level [112].

### Biological Scope

Cardiomyopathy, including heart failure with reduced ejection fraction (HFrEF) and HFpEF, is increasingly recognized as a multi-cellular reprogramming event rather than one driven by cardiomyocytes alone [113, 114]. Non-myocyte populations, including fibroblasts, endothelial cells, and immune cells such as macrophages, also undergo significant transcriptional and post-transcriptional remodeling during cardiac disease progression. The development of single-cell RNA sequencing has redefined our understanding of gene expression changes across diverse cardiac cell populations, revealing previously unappreciated cellular heterogeneity and gene expression patterns [115]. However, current literature has primarily focused on gene expression at the transcriptional level, with limited attention to alternative splicing regulation across different cell types in heart failure. For example, the aforementioned alternative splicing event of MyD88 has been shown to modulate NF-kB activation and cytokine production [33, 34]. In cardiac fibroblasts, TGF-β, a profibrotic signal, has been shown to regulate alternative splicing programs, leading to fibroblasts activation and extracellular matrix remodeling [116, 117]. Given the critical role of alternative splicing in cellular function beyond cardiomyocytes, an important future direction will be to extend splicing analyses beyond cardiomyocytes and systematically investigate splicing programs in non-cardiomyocyte populations. Single-cell splicing estimation algorithms, in combination with emerging technologies, provide a framework for linking splicing dynamics to cellular states and disease-associated cell populations. Integrating cell-type-specific splicing regulation into the multi-cellular models of cardiomyopathy will provide new insights into underlying disease mechanisms and yield novel therapeutic opportunities.

## Key References

- Wang ET, Sandberg R, Luo S, Khrebtukova I, Zhang L, Mayr C, Kingsmore SF, Schroth GP, Burge CB. Alternative isoform regulation in human tissue transcriptomes. Nature. 2008;456:470-476. 10.1038/nature07509.
  - ○ Findings fron this study identified extensive alternative splicing events in human tissues and potential tissue specificity of alternative splicing regulation.
- Lee JH, Gao C, Peng G, Greer C, Ren S, Wang Y, Xiao X. Analysis of transcriptome complexity through RNA sequencing in normal and failing murine hearts. Circ Res. 2011;109:1332-1341. 10.1161/circresaha.111.249433.
  - ○ Findings from this study identifeid differential alternative splicing in murine failing hearts.

## Data Availability

No datasets were generated or analysed during the current study.
